# Independent and combined associations of body mass index and visceral fat area with kidney function decline in a healthy Japanese urban population: a longitudinal study

**DOI:** 10.1186/s12882-025-04740-w

**Published:** 2026-01-08

**Authors:** Miwa Sonoda Enami, Aya Hirata, Kazuyo Kuwabara, Junji Miyazaki, Yoshimi Kubota, Yoko Nishida, Sachimi Kubo, Takumi Hirata, Tomoe Uchida, Aya Kadota, Aya Higashiyama, Daisuke Sugiyama, Tomofumi Nishikawa, Naomi Miyamatsu, Yoshihiro Miyamoto, Tomonori Okamura

**Affiliations:** 1 Department of Preventive Medicine and Public Health, Keio University School of Medicine, 35 Shinanomachi, Shinjuku-ku, Tokyo, 160-8582 Japan; 2 Department of Social Medicine, Graduate School of Medicine, Osaka University, Osakaï, 565-0871 Japan; 3 Department of Preventive Medicine, School of Medicine, Hyogo Medical University, Hyogo, 663-8501 Japan; 4 Osaka Institute of Public Health, Osaka, 537-0025 Japan; 5 Department of Public Health, Kindai University Faculty of Medicine, Osaka, 589- 8511 Japan; 6 Research Team for Human Care, Tokyo Metropolitan Institute for Geriatrics and Gerontology, Tokyo, 173-0015 Japan; 7 Department of Public Health, Shiga University of Medical Science, Shiga, 520-2192 Japan; 8 Department of Hygiene, Wakayama Medical University, Wakayama, 641-8509 Japan; 9 Faculty of Nursing and Medical Care, Keio University, Kanagawa, 252-0883 Japan; 10 Faculty of Health Science, Kyoto Koka Women’s University, Kyoto, 615-0822 Japan; 11 Department of Clinical Nursing, Shiga University of Medical Science, Shiga, 520- 2192 Japan; 12 Open Innovation Center, National Cerebral and Cardiovascular Center, Osaka, 564- 8565 Japan

**Keywords:** Obesity, Visceral fat, Chronic kidney disease, Longitudinal study

## Abstract

**Background:**

Obesity has been linked to progressive loss of kidney function, but the independent and combined associations of body mass index (BMI) and visceral fat accumulation remain uncertain in healthy individuals. This study aimed to clarify the longitudinal relationship between BMI, visceral fat area (VFA), and chronic kidney disease (CKD), defined as an estimated glomerular filtration rate based on serum cystatin C (eGFRcys) < 60 mL/min/1.73 m² among the Kobe Study participants targeting healthy urban residents in Japan.

**Methods:**

A total of 897 participants (267 men, 630 women) without CKD and with a mean age of 61.4 years were analyzed. VFA was measured using the impedance method, and participants were followed for a median of 8.3 years. Participants were classified into four groups based on baseline BMI (kg/m²) and VFA (cm²): G1 (VFA < 100 and BMI < 25), G2 (VFA < 100 and BMI ≥ 25), G3 (VFA ≥ 100 and BMI < 25), G4 (VFA ≥ 100 and BMI ≥ 25). Multivariable linear regression models were used to estimate adjusted mean annual changes in eGFRcys, and Cox proportional hazards models were applied to calculate hazard ratios (HRs) for incident CKD, adjusting for potential confounders (age, sex, smoking status, alcohol consumption, dyslipidemia, diabetes, hypertension and eGFRcys at baseline).

**Results:**

During follow-up, 85 participants (9.5%) developed CKD. The adjusted mean annual decline in eGFRcys was − 2.17% in G1, − 3.29% in G2, − 2.81% in G3, and − 3.81% in G4. Compared with G1, both G3 and G4 showed an elevated risks of CKD incidence (G3: HR 2.40, 95% CI 1.14–5.05; G4: HR 3.89, 95% CI 2.17–6.98), whereas the confidence interval crossed 1 for G2 (HR 0.88, 95% CI 0.12–6.47). Participants in G4 exhibited the steepest decline in kidney function and the highest CKD incidence rate (49.2 per 1,000 person-years).

**Conclusions:**

In healthy adults, the combination of elevated BMI and VFA was associated with accelerated kidney function decline. These findings underscore the importance of evaluating visceral fat in addition to BMI for early identification of individuals at increased risk of CKD and enable preventive strategies.

**Clinical trial number:**

Not applicable.

**Supplementary Information:**

The online version contains supplementary material available at 10.1186/s12882-025-04740-w.

## Background

Chronic kidney disease (CKD) is a major global public health challenge, and obesity has been consistently identified as a key risk factor for its development [1, 2]. While body mass index (BMI) is widely used to quantify obesity, it cannot distinguish between fat and lean mass or differentiate visceral from subcutaneous fat.

To address this, measures such as waist circumference (WC), waist-to-hip ratio, and visceral fat area (VFA) have been investigated as alternative or complementary indicators of harmful fat distribution [3]. VFA, in particular, is considered a more precise indicator of harmful adiposity, with previous studies showing its independent association with reduced glomerular filtration rate and incident CKD [4, 5]. Visceral fat plays a more direct role in metabolic dysfunction and CKD progression through mechanisms such as chronic inflammation, oxidative stress, and activation of the renin-angiotensin system [6]. A cross-sectional study among healthy Japanese urban residents found that greater visceral fat was associated with lower estimated GFR, even in a general population setting. Interestingly, in women, BMI showed a stronger association with renal function decline than VFA [7]. These findings suggest that BMI and VFA may have distinct or interactive associations, but longitudinal evidence from healthy populations remains scarce.

Recent evidence also highlights the utility of cystatin C–based estimated glomerular filtration rate (eGFRcys) in assessing renal function, especially in non-CKD populations. Unlike creatinine-based eGFR, eGFRcys is less influenced by muscle mass and more accurate in detecting early decline in kidney function [8, 9].

In particular, there is a need to better understand early-stage renal decline in middle-aged and older adults, who are at greater risk of developing CKD but may not yet meet clinical thresholds for intervention [10]. Moreover, middle-aged and older adults represent a critical target for early prevention, as lifestyle and metabolic changes during this life stage can accelerate organ decline [11].

Despite these insights, it remains unclear how general and visceral adiposity independently and jointly affect kidney function decline over time, particularly in Asian populations where visceral fat tends to accumulate more readily despite relatively low BMI. To fill this gap, we conducted a longitudinal study in a cohort of healthy urban Japanese adults aged 40–74 years to explore the earliest potential effects of visceral fat accumulation on declining kidney function. Using eGFRcys as a sensitive measure of renal function, we examine the individual and combined associations of BMI and VFA on CKD incidence, and identified subgroups at increased risk who may benefit from early preventive strategies.

## Objective

The present study aims to identify high risk populations of CKD by longitudinally investigating the associations between BMI and VFA with eGFRcys in the healthy urban cohort. This approach seeks to clarify the independent and combined associations of BMI and visceral fat accumulation on renal function decline, providing insights into early prevention and management strategies for CKD.

## Methods

### Study design and participants

This longitudinal study was part of the Kobe Orthopedic and Biomedical Epidemiological (KOBE) study, conducted between 2010 and 2020, with follow-up surveys every two years, and the KOBE NEXT study between 2022 and 2024. Participants of this cohort study were apparently healthy residents from an urban area in Kobe City in Japan. The primary objective of the study was to identify the incidence and causes of lifestyle-related diseases to support health maintenance and prevent a decline in quality of life. The inclusion criteria for participants were as follows: (1) aged 40 to 74 years, (2) no history of malignant neoplasm, cerebral disease, or cardiovascular disease, (3) not undergoing medication for hypertension, diabetes, or dyslipidemia, (4) self-reported good health, (5) ability to travel to the study site for required assessments, and (6) provided consent to participate in follow-up studies. Detailed methodologies and descriptions of these cohort studies have been published in previous research papers [12–14].

**Fig. 1:**
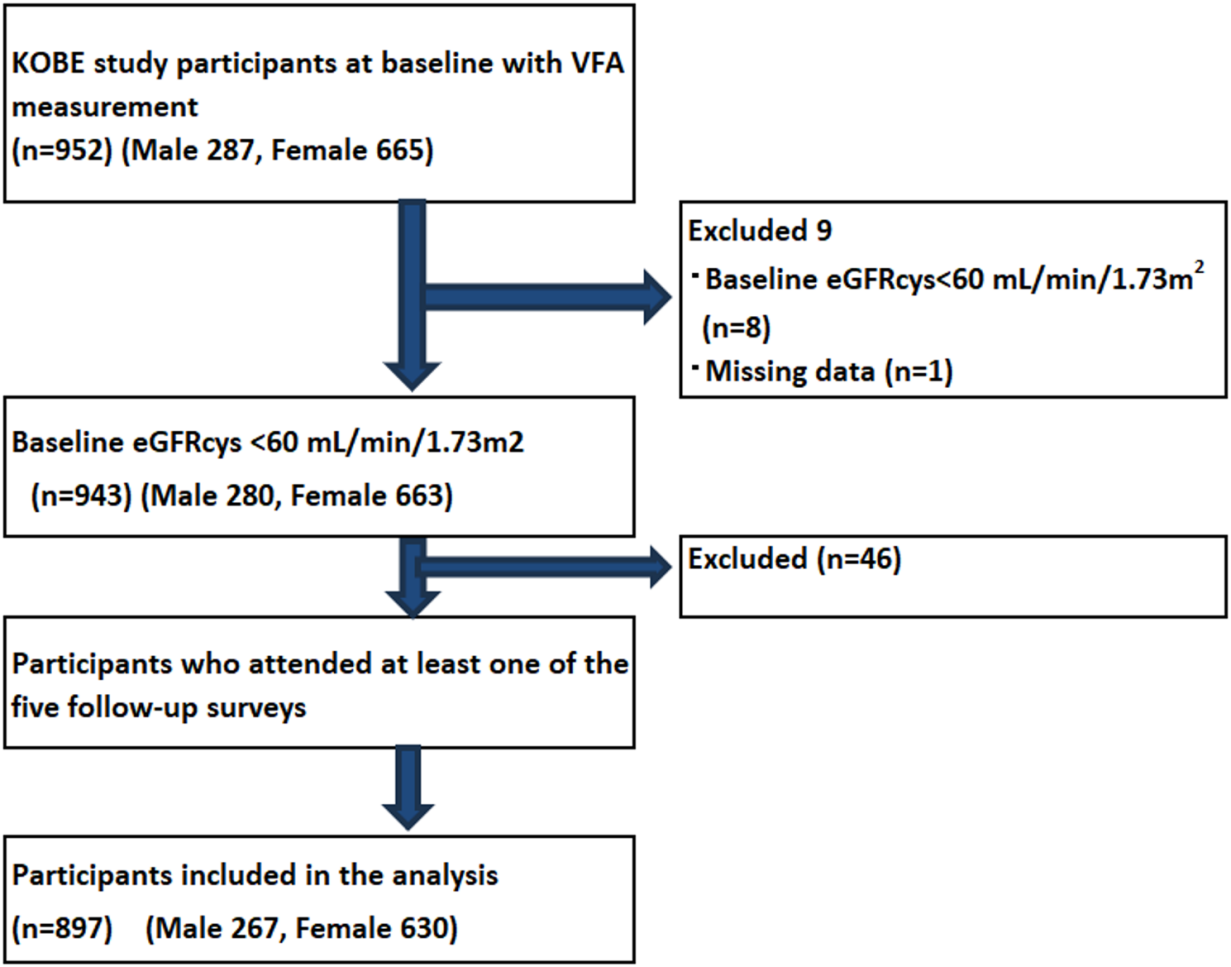
Flow diagram of patients included in this study

The participant flow diagram is presented in Fig. 1, and the study participants were selected as follows: Among the individuals who participated in the second follow-up of KOBE study conducted between 2014 and 2015, 952 had their visceral fat area (VFA) measured (287 men and 665 women); these individuals were defined as the baseline study population. After excluding 9 participants with baseline eGFRcys < 60 mL/min/1.73 m² (*n* = 8) or missing data (*n* = 1), 943 participants remained. Among these, 46 individuals who did not attend any of the five follow-up surveys were further excluded. Consequently, 897 participants (267 men and 630 women) were included in the final analysis.

### Ethical approval

This research has been approved by the Pharmaceutical Clinical Research Review Committee (Ethics Committee) of the Institute of Biomedical Research and Innovation at the Kobe Biomedical Innovation Cluster (approval no. 10–20) and the Ethics Committee of Keio University School of Medicine (approval no. 20170142 and 20211116). Participants were given written and oral explanations and provided us with written informed consent before study participation.

### Key variables and definitions

#### Measurement of eGFRcys

The following equation, tailored to the Japanese population, was used to calculate GRFcys. GFRcys = 104 × ScysC^− 1.019^ × 0.996^Age^ [× 0.929 if female] – 8 (mL = min = 1.73 m^2^) [15]. Furthermore, CKD was defined as eGFRcys < 60mL/min/1.73m^2^ [16]. Serum cystatin C levels were measured using a colloidal gold agglutination assay with the Nescote GC Cystatin C kit (Alfresa Pharma Corporation, Osaka, Japan).

#### Laboratory measurements

Height and body weight were measured with patients on stockinged feet and in light clothing, and BMI was calculated by dividing weight in kilograms by the squared height in meters. After a 5-min rest period, blood pressure was measured twice by an automatic sphygmomanometer (BP-103i II, Nihon Colin, Tokyo, Japan), and the mean value was used for analysis. Fasting blood samples were drawn from all participants after they had fasted for at least 10 h. Blood samples were transported to a single commissioned clinical laboratory center (SRL Inc., Tokyo, Japan). High blood pressure was defined as either receiving treatment for hypertension or having blood pressure reading of ≥ 130/80 mmHg [17]. Glucose intolerance was defined as either being treated for diabetes mellitus or having a fasting blood glucose level of ≥ 110 mg/dL or an HbA1c level of ≥ 6.0%, based on criteria for boarder type of diabetes mellitus from the Committee of the Japan Diabetes Society on the Diagnostic Criteria of Diabetes Mellitus [18]. Hypercholesterolemia was defined as either receiving treatment for elevated LDL-C or having an LDL-C level of ≥ 140 mg/dL, as outlined in the Japan Atherosclerosis Society Guidelines [19].

#### Visceral fat area

Visceral fat was measured using the VFA meter EW-FA90 (approved by the Pharmaceutical Affairs Law; Panasonic K.K., Osaka, Japan), which was based on the principle of the abdominal bioelectrical impedance analysis (BIA) method. This BIA method is noninvasive, allows for quick measurement, and demonstrates a strong correlation with Computed Tomography (CT) in estimating visceral fat accumulation [20]. Importantly, all VFA measurements were conducted by trained healthcare professionals. The measuring belt’s umbilical electrode unit was positioned at the participant’s umbilicus directly on the skin, and measurements were taken at the end of a normal exhalation in a standing position. Each measurement was repeated twice, and if the difference between the first two readings exceeded 5 cm², a third measurement was performed. The belts used for the measurement were selected based on the participant’s body size. Clinical trial results reported a correlation with CT imaging analysis (*r* = 0.88) [20].

#### Classification of obesity with visceral fat area and body mass index

The Japan Society for the Study of Obesity defines obesity as excessive fat accumulation with a BMI > 25 kg/m². Additionally, a VFA threshold of ≥ 100 cm² is considered to indicate visceral fat accumulation [21]. Participants were classified into four groups based on their obesity status using these criteria:

Group 1 (Normal group): VFA < 100 cm² and BMI < 25 kg/m².

Group 2 (Subcutaneous fat-type obese group): VFA < 100 cm² and BMI ≥ 25 kg/m².

Group 3 (Visceral fat-accumulated non-obese group): VFA ≥ 100 cm² and BMI < 25 kg/m².

Group 4 (Visceral fat-accumulated obese group): VFA ≥ 100 cm² and BMI ≥ 25 kg/m².

### Analytical methods

**Fig. 2:**
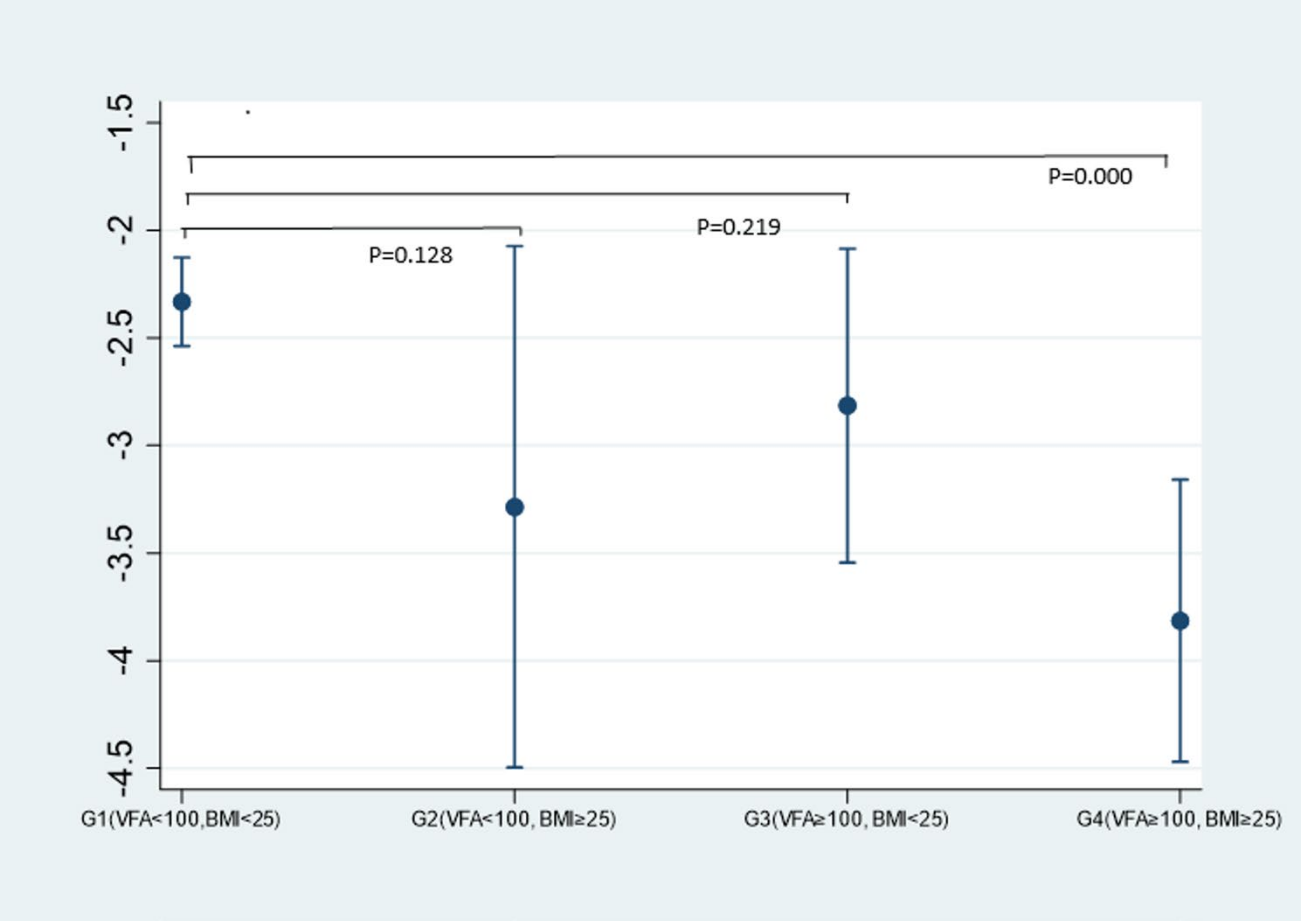
Annual percent change in eGFRcys across the four VFA-BMI groups. Error bars represent 95% confidence intervals. Adjusted using multivariable linear regression models for age, sex, alcohol consumption (current, past, or none), smoking status (current, past, or none), high blood pressure, hypercholesterolemia, glucose intolerance, and baseline eGFRcys. Sample sizes: G1 (*n* = 734), G2 (*n* = 21), G3 (*n* = 64), G4 (*n* = 78). BMI, Body Mass Index; CI, Confidence Interval; eGFRcys, estimated Glomerular Filtration Rate based on cystatin C; VFA, Visceral Fat Area

Participants’ baseline characteristics for continuous variables are presented as means (standard deviation: SD) or medians (interquartile range: IQR), while categorical variables were reported as frequency (%). To assess longitudinal changes in kidney function, multivariable linear regression models were applied to estimate adjusted mean annual changes in eGFRcys across the four VFA–BMI groups. In these models, the annual change in eGFRcys was defined as the dependent variable,, calculated as the adjusted mean change during the observation period divided by observation years, VFA–BMI groups were used as categorical dummy variables, and covariates including age, sex, alcohol consumption (never, past, or current), smoking status (never, past, or current), high blood pressure, hypercholesterolemia, glucose intolerance, and baseline eGFRcys were simultaneously adjusted. Adjusted marginal means and 95% confidence intervals (CIs) for each group were derived using the margins command in Stata, which calculates the expected outcome at each group level while averaging over the observed covariate distribution. For Fig. 2, these adjusted means were graphically displayed, and p-values for pairwise comparisons among the four groups were obtained within the framework of an analysis of covariance (ANCOVA) using the same multivariable regression model.

In addition, to evaluate the association between obesity indicators and the incidence of CKD during the follow-up period, Cox proportional hazards regression models were applied to estimate hazard ratios (HRs) and 95% confidence intervals (CIs). Three models were constructed: Model 1 adjusted for age, sex, and baseline eGFRcys; Model 2 additionally adjusted for alcohol consumption (never, past, or current), smoking status (never, past, or current), high blood pressure, hypercholesterolemia, and glucose intolerance; and Model 3 further adjusted for BMI in the VFA analyses or VFA in the BMI analyses. We also evaluated interaction terms between sex and the VFA–BMI groups to assess potential effect modification. In the Cox proportional hazards regression model, no interaction between sex and the VFA–BMI groups was identified (*p* = 0.151). The proportional hazards assumption was evaluated using Schoenfeld residuals. Neither the global test nor any variable-specific test indicated a significant violation (χ² = 18.87, *p* = 0.13 for Model 2), confirming that the proportional hazards assumption was satisfied in the multivariable Cox regression model. Kaplan–Meier curve was generated to visualize the hazard assumption across VFA–BMI groups.

Covariates were selected based on prior studies demonstrating their relevance to kidney function decline. In particular, age, diabetes, hypertension, and baseline eGFR were included as key clinical predictors [10, 22], while smoking and alcohol consumption were adjusted for as important modifiable lifestyle factors relevant to public health interventions [23].

Statistical analyses were conducted using STATA SE 17.0 (Stata Corp LP, College Station, TX, USA). A two-sided *p* < 0.05 was considered statistically significant. All confidence intervals were estimated.

### Result

### Baseline characteristics of study participants according to VFA/BMI stratification

The baseline characteristics of study participants according to the VFA-BMI groups are summarized in Table 1. A total of 897 participants were stratified into four VFA-BMI groups: 734 participants (81.8%) in G1, 21 (2.3%) in G2, 64 (7.1%) in G3, and 78 (8.7%) in G4. Overall, 630 participants (70.2%) were female. Females were predominant in the lower VFA groups, accounting for 77.7% in G1 and 90.5% in G2, whereas males were predominant in the higher VFA groups, with 82.8% in G3 and 61.5% in G4. Participants in the lowest VFA-BMI group (G1) exhibited lower levels of VFA, BMI, WC, as well as lower prevalence of high blood pressure prevalence, proportions of smokers (%) (current and past), compared to those in the other groups (G2-G4). Mean baseline eGFRcys was highest in G2 (96.9 mL/min/1.73 m²) and G1 (96.0 mL/min/1.73 m²), whereas participants in G3 and G4 showed lower mean values (88.3 and 84.2 mL/min/1.73 m², respectively). Mean WCdiffered by sex: 82.54 cm (95% CI 81.54–83.55) in men and 78.96 cm (95% CI 78.31–79.62) in women, with non-overlapping confidence intervals (data not shown). The correlation between baseline VFA and BMI is illustrated in Supplemental Fig. 1.

**Table 1:** Baseline characteristics of study participants according to VFA/BMI stratification

		G1(VFA < 100 and BMI < 25)	G2(VFA < 100 and BMI ≥ 25)	G3(VFA ≥ 100 and BMI < 25)	G4(VFA ≥ 100 and BMI ≥ 25)
		*N* = 734	*N* = 21	*N* = 64	*N* = 78
Sex, n(%)	Male	164 (22.3%)	2 (9.5%)	53 (82.8%)	48 (61.5%)
	Female	570 (77.7%)	19 (90.5%)	11 (17.2%)	30 (38.5%)
Age, years		62.5 (8.5)	58.1 (8.7)	64.3 (8.1)	64.6 (8.6)
VFA, cm²		50.9 (22.7)	83.9 (11.7)	119.0 (18.0)	134.8 (31.4)
BMI, kg/m²		20.6 (2.1)	26.3 (1.1)	23.3 (1.2)	27.3 (2.2)
WC, cm		77.5 (6.6)	88.4 (4.0)	87.1 (4.2)	95.5 (6.3)
High Blood pressure	No	633 (86.2%)	17 (81.0%)	43 (67.2%)	45 (57.7%)
	Yes	101 (13.8%)	4 (19.0%)	21 (32.8%)	33 (42.3%)
Hypercholesterolemia	No	418 (56.9%)	12 (57.1%)	40 (62.5%)	44 (56.4%)
	Yes	316 (43.1%)	9 (42.9%)	24 (37.5%)	34 (43.6%)
Glucose intolerance	No	628 (85.6%)	18 (85.7%)	50 (78.1%)	53 (67.9%)
	Yes	106 (14.4%)	3 (14.3%)	14 (21.9%)	25 (32.1%)
Smoking status	Never	598 (81.5%)	14 (66.7%)	24 (37.5%)	55 (70.5%)
	Past	116 (15.8%)	6 (28.6%)	33 (51.6%)	22 (28.2%)
	Current	20 (2.7%)	1 (4.8%)	7 (10.9%)	1 (1.3%)
Alcohol status	Never	352 (48.0%)	5 (23.8%)	51 (79.7%)	49 (62.8%)
	Past	21 (2.9%)	1 (4.8%)	1 (1.6%)	1 (1.3%)
	Current	361 (49.2%)	15 (71.4%)	12 (18.8%)	28 (35.9%)
eGFRcys (baseline)		96.0 (16.6)	96.9 (16.0)	88.3 (15.4)	84.2 (13.4)
Person-years of follow-up		4948.5	147.8	393.6	447.2

Data are presented as mean (SD) or median (IQR) for continuous measures, and n (%) for categorical measures. BMI, Body Mass Index; CKD, Chronic Kidney Disease; eGFRcys, estimated Glomerular Filtration Rate based on cystatin C; SD, Standard Deviation; VFA, Visceral Fat Area; WC, Waist circumference

### Baseline characteristics of study participants by CKD incidence

Table 2 summarizes the baseline characteristics of participants based on the incidence of CKD during the follow-up period. Among the 897 participants analyzed, 85 (9.5%) developed CKD. Participants who developed CKD were characterized by a higher proportion of males, older baseline age, higher VFA, higher BMI, larger WC, higher proportion of high blood pressure, greater numbers of current and past smokers, compared with those who did not develop CKD. In addition, individuals who developed CKD had substantially lower baseline eGFRcys and shorter follow-up duration.

**Table 2:** Baseline characteristics of participants grouped by incidence of CKD during the follow-up period

		Non-CKD (eGFRcys ≥ 60)	CKD (eGFRcys < 60)
		*N* = 812	*N* = 85
Sex, n(%)	Male	227 (28.0%)	40 (47.1%)
	Female	585 (72.0%)	45 (52.9%)
Age, years		62.0 (8.5)	69.7 (5.1)
VFA, cm²		61.1 (34.7)	89.2 (43.0)
BMI, kg/m²		21.3 (2.7)	23.2 (3.8)
WC, cm		79.5 (8.2)	84.9 (9.6)
High Blood pressure	No	680 (83.7%)	58 (68.2%)
	Yes	132 (16.3%)	27 (31.8%)
Hypercholesterolemia	No	469 (57.8%)	45 (52.9%)
	Yes	343 (42.2%)	40 (47.1%)
Glucose intolerance	No	676 (83.3%)	73 (85.9%)
	Yes	136 (16.7%)	12 (14.1%)
Smoking status	Never	638 (78.6%)	53 (62.4%)
	Past	148 (18.2%)	29 (34.1%)
	Current	26 (3.2%)	3 (3.5%)
Alcohol status	Never	413 (50.9%)	44 (51.8%)
	Past	21 (2.6%)	3 (3.5%)
	Current	378 (46.6%)	38 (44.7%)
eGFRcys (at baseline) mL/min/1.73 m2		96.6 (15.9)	74.5 (8.6)
eGFRcys (at last follow-up) mL/min/1.73 m2		84.4 (14.4)	56.2 (3.1)
Person-years of follow-up		5,549.8	387.3

Data are presented as mean (SD) or median (IQR) for continuous measures, and n (%) for categorical measures. BMI, Body Mass Index; CKD, Chronic Kidney Disease; eGFRcys, estimated Glomerular Filtration Rate based on cystatin C; SD, Standard Deviation; VFA, Visceral Fat Area; WC, Waist circumference

### Changes in eGFRcys across VFA-BMI groups

Table 3 presents the multivariable-adjusted means of eGFRcys and its changes over 8 years across the four VFA-BMI groups. At baseline, Group 4 (VFA ≥ 100 and BMI ≥ 25) showed the lowest baseline eGFRcys (88.7 mL/min/1.73 m²), whereas Group 1 (VFA < 100 and BMI < 25) had the highest (95.2 mL/min/1.73 m²). By the last follow-up, participants in Group 4 continued to show the lowest mean eGFRcys (77.2 mL/min/1.73 m²), while those in Group 1 had the highest (82.3 mL/min/1.73 m²).　 The greatest decline in kidney function was observed in Group 4, with an annual reduction of -3.41 mL/min/1.73 m²/year (95% CI: -3.98 to -2.84) and an annual percent change of -3.81% (95% CI: -4.47 to -3.16). Conversely, Group 1 experienced the smallest annual reduction, -2.17 mL/min/1.73 m²/year (95% CI: -2.35 to -1.99), with an annual percent change of -2.33% (95% CI: -2.54 to -2.13). Figure 2 illustrates the adjusted annual percent change in eGFRcys across the four VFA-BMI groups. Participants in Group 4 showed a greater decline in renal function compared with those in Group 1, with a clearly lower point estimate of annual percent change (− 3.81%/year vs. −2.33%/year). The 95% confidence intervals were narrow and did not overlap (95% CI: −4.47 to − 3.16 vs. −2.54 to − 2.13), indicating a precise and consistent difference in the direction of steeper eGFRcys decline.

**Table 3:** Multivariable-adjusted means of eGFRcys and its changes over the follow-up period across the four VFA-BMI groups

	G1 (VFA < 100 and BMI < 25)	G2 (VFA < 100 and BMI ≥ 25)	G3 (VFA ≥ 100 and BMI < 25)	G4 (VFA ≥ 100 and BMI ≥ 25)
	*N* = 734	*N* = 21	*N* = 64	*N* = 78
	Mean (95%CI)	Mean (95%CI)	Mean (95%CI)	Mean (95%CI)
eGFRcys (at baseline), mL/min/1.73 m2	95.18 (94.28, 96.08)	90.43 (85.15, 95.72)	94.51 (91.32, 97.69)	88.72 (85.88, 91.56)
eGFRcys (at last follow-up), mL/min/1.73 m2	82.32 (81.66, 82.98)	79.94 (76.04, 83.85)	80.53 (78.18, 82.89)	77.24 (75.12, 79.35)
eGFRcys amount of change, mL/min/1.73 m2	-12.14 (-12.80, -11.47)	-14.51 (-18.42, -10.60)	-13.92 (-16.28, -11.57)	-17.22 (-19.34, -15.10)
eGFRcys amount of change, mL/min/1.73 m2/year	-2.17 (-2.35, -1.99)	-3.18 (-4.22, -2.13)	-2.64 (-3.27, -2.01)	-3.41 (-3.98, -2.84)
Annual percent change in eGFRcys %/year	-2.33 (-2.54, -2.13)	-3.29 (-4.50, -2.07)	-2.81 (-3.54, -2.09)	-3.81 (-4.47, -3.16)

Adjusted for age, sex, alcohol consumption (current, past, or none), smoking status (current, past, or none), high blood pressure, hypercholesterolemia, glucose intolerance and baseline eGFRcys. BMI, Body Mass Index; CI, Confidence Interval; eGFRcys, estimated Glomerular Filtration Rate based on cystatin C; VFA, Visceral Fat Area

### Risk of CKD incidence by different obesity indicators

Table 4 presents the incidence rates and multivariable-adjusted hazard ratios (HRs) for CKD development according to obesity indicators.

Visceral Fat Area (VFA): Participants with VFA ≥ 100 cm² had a markedly higher incidence rate of CKD (40.4 per 1,000 person-years) compared with those with VFA < 100 cm² (10.0 per 1,000 person-years). The multivariable-adjusted HRs for participants with VFA ≥ 100 cm² were consistently higher than those with VFA < 100 cm², ranging from 2.57 to 3.45 across models, with relatively narrow 95% confidence intervals (e.g., 95% CI: 2.10–5.67 in Model 1), suggesting a robust and precise positive association between higher visceral fat and CKD incidence.

Body Mass Index (BMI): Participants with BMI ≥ 25 kg/m² also had a higher incidence rate of CKD (38.7 per 1,000 person-years) than those with BMI < 25 kg/m² (11.6 per 1,000 person-years). The adjusted HRs were 3.51 (95% CI: 2.14–5.76) in Model 1, 2.71 (95% CI: 1.62–4.55) in Model 2, and 1.48 (95% CI: 0.75–2.91) in the VFA-adjusted Model 3.

Combined VFA-BMI Groups: Among the four combined groups, Group 4 (VFA ≥ 100 cm² and BMI ≥ 25 kg/m²) showed the highest risk, with HRs around fourfold higher than Group 1 and relatively tight confidence intervals, with HRs of 4.61 (95% CI: 2.70–7.87) in Model 1 and 3.89 (95% CI: 2.17–6.98) in Model 2, indicating a strong and precise association. Group 3 (VFA ≥ 100 cm² and BMI < 25 kg/m²) also demonstrated elevated HRs, 2.18 (95% CI: 1.08–4.37) in Model 1 and 2.40 (95% CI: 1.14–5.05) in Model 2. In contrast, Group 2 (VFA < 100 cm² and BMI ≥ 25 kg/m²) had an HR close to 1, with wide confidence intervals (reflecting small sample size) and overlapping unity, indicating no clear association with CKD risk.

**Table 4:** Incidence rates and multivariable-adjusted hazard ratios (95% CI) for CKD development by obesity indicators

	*N*	Person Years (PY)	CKD Incidence	CKD Incidence rate (/1000PY)	Model1	Model2	Model3
Visceral fat							
VFA < 100 cm²	755	5096.3	51	10.0	1.0 (Ref)	1.0 (Ref)	1.0 (Ref)
VFA ≧ 100 cm²	142	840.8	34	40.4	3.45 (2.10–5.67)	3.29 (1.91–5.65)	2.57 (1.28–5.16)
BMI							
BMI < 25 kg/m²	798	5342.1	62	11.6	1.0 (Ref)	1.0 (Ref)	1.0 (Ref)
BMI ≧ 25 kg/m²	99	595.0	23	38.7	3.51 (2.14–5.76)	2.71 (1.62–4.55)	1.48 (0.75–2.91)
VFA-BMI groups							
G1(VFA < 100 and BMI < 25)	734	4948.5	50	10.1	1.0 (Ref)	1.0 (Ref)	NA
G2(VFA < 100 and BMI > = 25)	21	147.8	1	6.8	1.19 (0.16–8.64)	0.88 (0.12–6.47)	NA
G3(VFA > = 100 and BMI < 25)	64	393.6	12	30.5	2.18 (1.08–4.37)	2.40 (1.14–5.05)	NA
G4(VFA > = 100 and BMI > = 25)	78	447.2	22	49.2	4.61 (2.70–7.87)	3.89 (2.17–6.98)	NA

Hazard ratios were derived from Cox proportional hazards regression models. Model 1 was adjusted for age, sex and baseline eGFRcys; Model 2 included all variables from Model 1, with additional adjustment for alcohol consumption (never, past, or current), smoking status (never, past, or current), high blood pressure, hypercholesterolemia, and glucose intolerance; Model 3 included all variables from Model 2, with BMI added as an adjustment variable for VFA analyses, and VFA added as an adjustment variable for BMI analyses

## Discussion

In an apparently healthy urban population, a total of 897 participants were categorized into four VFA-BMI groups, with 9.5% (*N* = 85) developing CKD during the follow-up period. In the present study, the adjusted mean eGFRcys decline among all participants was − 2.34 mL/min/1.73 m²/year (SD: 2.54), with G4 (VFA ≥ 100 and BMI ≥ 25) showing the highest decline at − 3.41 mL/min/1.73 m²/year (95% CI: − 3.98 to − 2.84), corresponding to − 3.81% per year.

### Comparison with previous studies

Compared to previous studies, the eGFRcys decline observed in our study population, particularly among participants with both high BMI and high VFA, was substantially greater even in the absence of CKD at baseline, emphasizing the potential risks associated with excess adiposity. For instance, a nationwide study among Japanese health check-up participants aged ≥ 40 years reported an average eGFR decline of -0.36 mL/min/1.73 m²/year, much lower than the mean eGFR decline of our study participants [24]. Another single-center study in CKD patients found a decline rate of -1.64 ± 0.48 mL/min/1.73 m²/year; within that cohort, individuals in the highest BMI quartile experienced a faster decline (-3.40 vs. -1.75 mL/min/1.73 m²/year) [25]. These findings indicate a consistent association between obesity and renal function decline. However, our study is novel in that it shows a similar or even greater decline rate in a non-CKD general population, suggesting that excess visceral fat and elevated BMI may contribute to earlier renal deterioration even before clinical CKD onset.

In the present study, when we stratified by both VFA and BMI, individuals who had both VFA ≥ 100 cm² and BMI ≥ 25 kg/m² demonstrated both a greater annual decline in eGFRcys (− 3.81%/year; 95% CI: −4.47 to − 3.16) and a higher CKD incidence (HR 3.89; 95% CI: 2.17–6.98) compared with the reference group. Our previous cross-sectional study demonstrated an association between VFA and renal function decline among healthy individuals, but also reported that in women, BMI had a stronger association with renal function decline than VFA [7]. Similarly, Bullen et al. reported that indices of visceral fat, such as Lipid Accumulation Product and Visceral Adiposity Index, were associated with increased risk of new-onset CKD but did not provide additional predictive value beyond WC and BMI [3]. However, previous research has also highlighted the role of visceral fat in metabolic risk and renal function decline. The Framingham Study demonstrated that visceral adipose tissue (VAT) was more strongly associated with an unfavorable metabolic risk profile than subcutaneous adipose tissue (SAT), emphasizing the importance of visceral fat in metabolic health [2]. Madero’s longitudinal study in elderly individuals found a direct association between VFA and renal function decline, suggesting that visceral fat contributes to CKD progression over time [4]. Additionally, Manabe et al. observed that for every 10-unit increase in VFA, the risk of CKD progression increased, showing a stronger association than BMI and WC, particularly among individuals with VFA < 100 cm² [26]. Kataoka et al. further demonstrated that a higher VAT/SAT ratio was linked to renal function decline, particularly in CKD patients with VFA < 100 cm². These findings highlight that while BMI remains a strong predictor, visceral fat also plays an independent role in CKD progression [5]. A previous Japanese study reported that low HDL-C and metabolic syndrome, but not general obesity, were associated with eGFR decline during a short one-year follow-up period [27]. In contrast, our study identified both high BMI and increased VFA as significant predictors of renal function decline over an extended eight-year follow-up. This difference in follow-up duration is a key factor: while short-term studies may reflect transient or reversible metabolic associations, longer follow-up enables the detection of progressive kidney damage from chronic adiposity, such as glomerular hypertrophy and interstitial fibrosis. Differences in baseline health status may also contribute, as the previous study included participants with treated hypertension, diabetes, and dyslipidemia, whereas our study excluded such comorbidities at the time of cohort enrollment, allowing for a more isolated assessment of adiposity-related effects.

### Biological mechanisms and clinical implications

The mechanisms by which BMI and visceral fat contribute to CKD have been widely studied. An elevated BMI may lead to CKD through glomerular hyperfiltration, inflammation, and neurohormonal activation [1].Visceral fat, in particular, is associated with chronic inflammation and oxidative stress, which promote insulin resistance, adipocyte dysfunction, and renal function deterioration [3, 6, 28]. It also secretes elevated levels of inflammatory adipocytokines, including MCP-1, TNF-α, PAI-1, and IL-6, contributing to systemic inflammation, and microvascular damage [29, 30]. Japanese individuals tend to store less subcutaneous fat compared to their Western counterparts, leading to more visceral fat accumulation in organs such as the kidneys and liver [31]. This ectopic fat may accelerate CKD progression by promoting fibrosis and metabolic dysfunction. Given the role of visceral fat in CKD and cardiovascular disease, assessing it independently of BMI and WCis justified [29]. Our findings suggest that the combination of BMI and VFA is associated with the greater risk of incident CKD than either measure alone. These pathophysiologic processes may explain the substantial eGFR decline observed over time, even in the absence of overt CKD at baseline. In fact, D’Agati et al. reported that obesity-related glomerulopathy is characterized by glomerulomegaly and that mild renal pathological alterations often occur in individuals with morbid obesity, even before clinical kidney disease is diagnosed [32]. Based on these findings, a screening approach using both BMI and VFA may improve CKD risk stratification. Further studies are needed to elucidate the underlying mechanisms by which the combination of visceral and general obesity contributes to accelerated renal decline.

### Strengths and limitations

This study has several strengths. First, by analyzing a healthier population with relatively low BMI and non-obesity, this study could minimize confounding factors such as general obesity or related comorbidities as well as help identify individuals at higher risk of early renal function decline, even within a pre-disease state. This study may provide a clearer understanding of the associations of visceral fat accumulation and BMI with renal function decline over time. Second, using cystatin C to calculate eGFR allowed for a more accurate assessment of renal function compared to creatinine-based methods, as this method is minimally influenced by factors such as gender, age, and muscle mass [15]. However, this study has also several limitations. First, in the present study, CKD was defined as a single measurement of eGFRcys < 60 mL/min/1.73 m² at each point. Because data was obtained from health checkups conducted every two years, we could not confirm whether reduced eGFRcys persisted for at least 3 months [16], and thus the chronicity criterion for CKD diagnosis could not be assessed. Thus, it may have caused misclassification. As this cohort lacked data on proteinuria, we could not account for incident proteinuria in identifying new CKD incident. Consequently, the overall CKD incidence might have been underestimated. Second, due to the limited number of CKD cases—particularly among men—we were unable to conduct sex-stratified analyses. Instead, we adjusted for sex as a covariate in all models. Although previous cross-sectional studies suggest sex-specific associations between visceral fat and eGFRcys [7], our longitudinal design and limited sample size precluded a detailed examination. Third, one important limitation of this study is the limited statistical power for the comparison between G1 and G2 due to the very small number of participants in G2. Consequently, the results for this subgroup should be interpreted with caution and may not be robust. Fourth, VFA and BMI were only assessed at baseline, and changes in obesity status over the follow-up period were not accounted for. It is possible that some participants may have changed categories during the study period, which could have affected the observed associations. Future studies incorporating time-varying exposures are needed to address this limitation. Fifth, participants without follow-up measurements were excluded from the analysis. Some of these exclusions were due to disruptions in follow-up during the COVID-19 pandemic. A marked decline in follow-up surveys was observed in fiscal year 2020 (332 participants) compared with previous survey waves (897, 877, and 825 participants at baseline, the 1st and 2nd follow-ups, respectively), likely reflecting the impact of the COVID-19 pandemic on voluntary participation in health examinations. Participation partially recovered in 2023 (532 participants). Sixth, mortality data were not available in this study because the cohort consisted of participants who voluntarily attended each follow-up health examination. Although some participants lost to follow-up may have included deceased individuals, the observation period for each participant was defined up to the last confirmed follow-up date. Therefore, selection bias cannot be completely ruled out, and the baseline characteristics of those who did not participate in any follow-up examinations are presented in Supplementary Table 1. Seventh, the study was performed in a Japanese urban city, which may limit the generalizability of our findings to other populations, such as rural residents. Lastly, although the device used to measure VFA by the BIA method demonstrates a strong correlation with the values measured by X-ray CT [20], we did not obtain CT scans from participants to directly compare BIA-derived VFA with CT-based measurements. Because this cohort consisted of very healthy community residents, X-ray-based examinations were deemed highly invasive at the time of study planning.

## Conclusion

In this longitudinal study of apparently healthy urban residents, both elevated BMI and increased VFA were associated with kidney function decline and higher incidence of CKD. Notably, participants with both high VFA and high BMI (G4) exhibited the steepest decline and the greatest risk, and those with high VFA but normal BMI (G3) also demonstrated an increased risk of CKD. These findings suggest that VFA, a marker of visceral fat accumulation, provides additional predictive value upon BMI in identifying individuals at higher risk for future kidney function deterioration, even in a non-CKD population. Therefore, combining VFA with BMI might enhance risk stratification and enable earlier identification of at-risk individuals. Importantly, these findings are derived from a Japanese cohort, and the degree to which they generalize to populations with different ethnic or lifestyle backgrounds remains to be determined. Further studies with diverse populations, long-term follow-up data, and larger sample sizes are warranted.

## Supplementary Information

Below is the link to the electronic supplementary material.

Supplementary Material 1

## Data Availability

The datasets generated and analyzed during the current study are not publicly available due to institutional data use agreements and participant confidentiality but are available from the corresponding author on reasonable request.

## Abbreviations

BMI: Body Mass Index
BIA: Bioelectrical Impedance Analysis
CKD: Chronic Kidney Disease
CI: Confidence Interval
CT: Computed Tomography
eGFR: Estimated Glomerular Filtration Rate
eGFRcys: Estimated Glomerular Filtration Rate based on Serum Cystatin C
HbA1c: Hemoglobin A1c
IQR: Interquartile Range
KOBE: Kobe Orthopedic and Biomedical Epidemiological Study
LDL-C: Low-Density Lipoprotein Cholesterol
LAP: Lipid Accumulation Product
PAI-1: Plasminogen Activator Inhibitor-1
SAT: Subcutaneous Adipose Tissue
SD: Standard Deviation
TNF-α: Tumor Necrosis Factor Alpha
VAI: Visceral Adiposity Index
VAT: Visceral Adipose Tissue
VFA: Visceral Fat Area
WC: Waist Circumference
