# Supplementary material for: Independent and combined associations of body mass index and visceral fat area with kidney function decline in a healthy Japanese urban population: a longitudinal study
Source: BMC Nephrol. 2026 Jan 8;27:96. doi: 10.1186/s12882-025-04740-w (PMC12882207; doi:10.1186/s12882-025-04740-w)
Supplement: Supplementary file 1 — Supplementary Material 1 [file 12882_2025_4740_MOESM1_ESM.docx]

**Supplementary Table 1. Baseline characteristics of study participants who did not participate in any follow-up survey, according to VFA/BMI stratification**

|  |  | G1(VFA < 100 and BMI < 25) | G2(VFA<100 and　BMI>=25) | G3(VFA>=100 and BMI<25) | G4(VFA>=100 and BMI>=25) |
| --- | --- | --- | --- | --- | --- |
|  |  | N=39 | N＝0 | N=5 | N=2 |
| Sex, n(%) | Male | 9 (23.1%) |  | 4 (80.0%) | 0 (0.0%) |
|  | Female | 30 (76.9%) |  | 1 (20.0%) | 2 (100.0%) |
| Age, years |  | 65.6 (8.6) |  | 67.0 (10.3) | 68.0 (11.3) |
| VFA, cm² |  | 53.8 (24.9) |  | 114.1 (11.4) | 111.5 (13.4) |
| BMI, kg/m² |  | 20.4 (2.3) |  | 23.2 (1.3) | 26.4 (1.6) |
| Waist circumference, cm |  | 78.3 (6.7) |  | 86.3 (6.0) | 95.8 (2.5) |
|  |  |  |  |  |  |
| High Blood pressure | No | 35 (89.7%) |  | 1 (20.0%) | 2 (100.0%) |
|  | Yes | 4 (10.3%) |  | 4 (80.0%) | 0 (0.0%) |
| Hypercholesterolemia | No | 20 (51.3%) |  | 3 (60.0%) | 1 (50.0%) |
|  | Yes | 19 (48.7%) |  | 2 (40.0%) | 1 (50.0%) |
| Glucose intolerance | No | 30 (76.9%) |  | 2 (40.0%) | 1 (50.0%) |
|  | Yes | 9 (23.1%) |  | 3 (60.0%) | 1 (50.0%) |
| Smoking status | Never | 32 (82.1%) |  | 1 (20.0%) | 2 (100.0%) |
|  | Past | 6 (15.4%) |  | 3 (60.0%) | 0 (0.0%) |
|  | Current | 1 (2.6%) |  | 1 (20.0%) | 0 (0.0%) |
| Alcohol status | Never | 16 (41.0%) |  | 4 (80.0%) | 1 (50.0%) |
|  | Past | 3 (7.7%) |  | 0 (0.0%) | 0 (0.0%) |
|  | Current | 20 (51.3%) |  | 1 (20.0%) | 1 (50.0%) |
| eGFRcys (baseline) |  | 91.3 (15.2) |  | 81.9 (10.3) | 82.6 (1.2) |

Data are presented as mean (SD) or median (IQR) for continuous measures, and n (%) for categorical measures. BMI, Body Mass Index; CKD, Chronic Kidney Disease;

eGFRcys, estimated Glomerular Filtration Rate based on cystatin C; SD, Standard Deviation; VFA, Visceral Fat Area.

**Supplemental Figure1　 Correlation between baseline VFA and BMI**
